# Increased glutamate in type 2 diabetes in the Korean population is associated with increased plasminogen levels

**DOI:** 10.1111/1753-0407.13429

**Published:** 2023-06-14

**Authors:** Hyo Jung Lee, Jeong Won Yeom, Ji Ho Yun, Han Byul Jang, Min‐Gyu Yoo, Hyo‐Jin Kim, Soo Kyung Koo, Hye‐Ja Lee

**Affiliations:** ^1^ Division of Endocrine and Kidney Disease Research, Department of Chronic Disease Convergence Research Korea National Institute of Health, Korea Disease Control and Prevention Agency Cheongju‐si Chungcheongbuk‐do Korea

**Keywords:** glutamate, Korean Genome and Epidemiology Study, metabotropic glutamate receptor 5, plasminogen, plasminogen activator inhibitor‐1, type 2 diabetes, 谷氨酸, 韩国基因组和流行病学研究, 代谢性谷氨酸受体5, 纤溶酶原, 纤溶酶原激活物抑制剂‐1, 2型糖尿病

## Abstract

**Background:**

Glutamate is a major neurotransmitter, although it causes cytotoxicity and inflammation in nonneuronal organs. This study aimed to investigate the metabolic disorders in which glutamate, associated with type 2 diabetes onset, is induced in the liver.

**Methods:**

An analysis of Korean community‐based Ansan‐Ansung cohort study data as well as functional research using in vitro and mouse models were performed.

**Results:**

Groups with high plasma glutamate levels (T2, T3) had a significantly increased risk of diabetes incidence after 8 years, compared to the group with relatively low glutamate levels (T1). Analysis of the effect of glutamate on diabetes onset in vitro showed that glutamate induces insulin resistance by increasing glucose‐related protein 78 (GRP78) and phosphoenolpyruvate carboxykinase (PEPCK) expression in SK‐Hep‐1 human liver cells. In addition, three different genes, *FRMB4B*, *PLG*, and *PARD3,* were significantly associated with glutamate and were identified via genome‐wide association studies. Among glutamate‐related genes, plasminogen (PLG) levels were most significantly increased in several environments in which insulin resistance was induced, and was also upregulated by glutamate. Glutamate‐induced increase in PLG in liver cells was caused by metabotropic glutamate receptor 5 activation, and PLG levels were also upregulated after extracellular secretion. Moreover, glutamate increased the expression of plasminogen activator inhibitor‐1 (PAI‐1). Thus, extracellular secreted PLG cannot be converted to plasmin (fibrinolytic enzyme) by increased PAI‐1.

**Conclusions:**

Increased glutamate is closely associated with the development of diabetes, and it may cause metabolic disorders by inhibiting the fibrinolytic system, which plays an important role in determining blood clots, a hallmark of diabetes.

## Graphical abstract

**Highlights**

- Increased glutamate is associated with the development of diabetes.
- Glutamate increases plasminogen (PLG) levels through glutamate metabotropic receptor 5 activation with various insulin resistance‐related stresses.
- Glutamate inhibits the conversion of PLG to plasmin by increasing plasminogen activator inhibitor‐1.

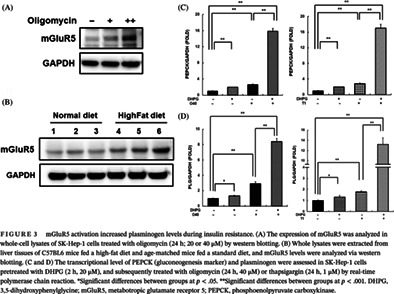

## 摘要

背景:谷氨酸是一种主要的神经递质, 尽管它在非神经元器官中引起细胞毒性和炎症。本研究旨在探讨与2型糖尿病发病相关的谷氨酸在肝脏中诱导的代谢紊乱。

方法:对韩国基于社区的Ansan‐Ansung队列研究数据进行分析, 并利用体外和小鼠模型进行功能研究。

结果:与谷氨酸水平相对较低组(T1)相比, 高谷氨酸水平组(T2, T3)8年后糖尿病发病风险显著增加。谷氨酸对糖尿病发病影响的体外研究显示, 谷氨酸通过增加人肝细胞SK‐Hep‐1中葡萄糖相关蛋白78 (GRP78)和磷酸烯醇式丙酮酸羧激酶(PEPCK)的表达诱导胰岛素抵抗。此外, 三个不同的基因FRMB4B, PLG和PARD3, 通过全基因组关联研究被鉴定出与谷氨酸显著相关。在与谷氨酸相关的基因中, 纤溶酶原(PLG)水平在几种诱导胰岛素抵抗的环境中显著升高, 并且调节谷氨酸。谷氨酸诱导的肝细胞PLG增加是由代谢型谷氨酸受体5激活引起的, 细胞外分泌后PLG水平也上调。此外,谷氨酸还增加纤溶酶原激活物抑制剂‐1 (PAI‐1)的表达。因此, 细胞外分泌的PLG不能被升高的PAI‐1转化为纤溶酶。

## 1 INTRODUCTION

Type 2 diabetes is a global health problem and a chronic disease that shortens the human life span by causing various complications.^1^ Insulin resistance, one of the strongest predictive factors for diabetes, is found in approximately 80%–90% of patients with type 2 diabetes, and it causes functional disorders associated with insulin receptors, abnormal insulin signaling, and abnormal glucose metabolism, which subsequently cause diabetes.^2^, ^3^, ^4^, ^5^

In particular, insulin resistance causes various metabolic abnormalities such as changes in lipid levels, hyperglycemia, and inhibition of fibrinolysis.^6^ Reportedly, a decrease in fibrinolytic ability is a characteristic of diabetes with insulin resistance and plays an important role in determining thrombosis.^2^, ^7^, ^8^ The fibrinolytic system begins with the co‐localization of plasminogen (PLG) and tissue plasminogen activator (tPA) with fibrin. PLG, a component that initiates fibrinolysis, is an enzyme synthesized in the liver, and its activation can break down insoluble fibrin.^9^ However, post‐translational modifications, deficiency, and restriction of activation of PLG can cause vascular obstruction and clots in the liver, stomach, pancreas, and lungs.^8^ Moreover, studies have shown that plasminogen activator inhibitor‐1 (PAI‐1), a major physiological inhibitor of plasminogen activators (PAs), is an important factor in limiting fibrinolytic activity.^7^, ^10^, ^11^ Increased PAI‐1 levels inhibit the action of PAs and induce blood clotting.^10^, ^11^ In the setting of insulin resistance and diabetes, a thrombotic milieu is created by an increase in hepatic synthesis of prothrombotic factors, including PAI‐1.^7^, ^8^

As explained previously, the basis of metabolic disorders is an imbalance in the metabolic control system. Amino acid and protein metabolism are abnormal in conditions of insulin resistance, diabetes, and obesity.^12^ Recently, several metabolite studies have shown that glutamate concentration is increased in diabetic patients, indicating that glutamate function is associated with the onset of type 2 diabetes.^13^, ^14^ Although glutamate is a neurotransmitter, it is a proteinogenic amino acid secreted by pancreatic α‐cells and performs various functions in peripheral organs, such as the liver, kidney, lung, and heart.^15^, ^16^ Glutamate and its receptors induce cytotoxicity, which is implicated in the control of inflammatory reactions and fibrosis in a few non‐neurological diseases.^16^, ^17^ In particular, the liver is a major source of plasma glutamate.^16^ High doses of glutamate can directly induce hepatotoxicity in vivo and exogenous glutamate injection can induce severe liver inflammation.^18^, ^19^, ^20^ Hyperglutamatemia in diabetes is caused by abnormal protein metabolism in the liver and muscles and via immoderate glutamate release by activated platelets.^12^

The effects of abnormal glutamate and its receptors and regulating enzymes on diabetes are being studied. Reportedly, glutamate induces retinal function loss through the activation of its metabotropic glutamate receptor 5 (mGluR5) receptor, and the abnormal activity of multiple mGluRs affects diabetic neuropathy development in a diabetic rat model.^21^, ^22^ However, studies on the metabolic effects of glutamate and its receptors on the liver in relation to the development of diabetes are insufficient.

In this study, we aimed to evaluate the association between diabetes onset and glutamate levels using Korean community‐based cohort data. We performed a genome‐wide association study (GWAS) to identify modulators involved in the onset of glutamate‐related diabetes. Further, we analyzed the effect of glutamate on the liver via its receptors and regulators in relation to diabetes onset using in vitro and mouse models.

## 2 METHODS

### 2.1 Study population

This study was approved by the Institutional Review Board of the National Biobank of Korea and the Korea National Institute of Health (2017‐02‐06‐P‐A). The study procedures were carried out in accordance with the approved guidelines. Data were obtained from the Ansan‐Ansung cohort study conducted by the Korea National Institute of Health as part of the Korean Genome and Epidemiology Study (KoGES). A total of 10 030 individuals aged 40–69 years were recruited between 2001 and 2014 and followed up using a survey every 2 years.^23^ In this study, we used data from the second (2005–2006) to the sixth follow‐up (2013–2014). For 2580 individuals whose metabolite data were available (AbsoluteIDQ™ p180 Kit),^24^ subjects with diabetes at baseline (*n* = 586) and those with insufficient data on diabetes (baseline = 13, follow‐up = 137) were excluded from the study (Figure S1). Diabetes was defined using at least one of the following criteria: self‐reported diagnosed diabetes, treatment with hypoglycemic medication, fasting glucose levels of ≥126 mg/dL, or plasma glucose levels of ≥200 mg/dL after the oral glucose tolerance test.

### 2.2 Statistical analysis

Statistical analyses were performed using PLINK (ver. 1.9) and SAS software (ver. 9.4.; SAS Institute Inc.). Variables with non‐normal distributions were log‐transformed before the analysis. To evaluate differences in characteristics associated with glutamate levels, the subjects were classified into three groups according to glutamate levels. T3 indicates the group with the highest plasma glutamate tertile, and the differences among the three groups were assessed using a general linear model or chi‐square test. The association between glutamate levels and the incidence of diabetes was assessed using a Cox proportional hazards regression model that included age and sex as covariates. Kaplan–Meier estimates of the cumulative probability of incident diabetes over time were assessed. Screening of genetic variants for plasma glutamate levels was performed using linear regression analysis after adjusting for age and sex. Genotype data for the Ansan‐Ansung cohort study were provided by the National Biobank of Korea and the Centers for Disease Control and Prevention. Genotyping was performed using the Affymetrix Genome‐Wide Human SNP Array (ver. 5.0; Affymetrix Inc.). Genotype imputation was performed using IMPUT2 and 10 000 genome projects of East Asian ancestry samples that were used as a reference panel.^25^, ^26^

### 2.3 Cell culture

SK‐Hep‐1 human liver cells were purchased from the American Type Culture Collection (ATCC). The cells were maintained in DMEM supplemented with 10% FBS, 100 units/mL penicillin, and 100 μg/mL streptomycin. All cell culture supplements were purchased from GIBCO (Thermo Fisher Scientific Inc.). The growth medium was replaced every 2 days, and cells were maintained at subconfluence in 95% air and 5% CO_2_ in a humidified atmosphere at 37°C.

### 2.4 Chemical treatment

SK‐Hep‐1 cells (5 × 10^5^) were seeded onto 60 mm dishes, incubated for 24 h, and treated with the indicated chemicals according to the manufacturer's recommendations. L‐Glutamic acid monosodium salt monohydrate (MSG) was dissolved in cell‐culture grade water and used at 10–40 mM in low‐glucose DMEM with 2% FBS for 24 h. Thapsigargin was dissolved in DMSO and 0.3 to 1 μM was used in culture media with 2% FBS for 24 h. Oligomycin was dissolved in ethanol and incubated at 20–40 μM in culture media for 24 h. 3,5‐dihydroxyphenylglycine (DHPG; 20 μM) was dissolved in DMSO and added 2 h before thapsigargin or oligomycin treatment. Palmitate (PA)‐BSA solution was added at a concentration of 500 μM for 24 h. To prepare the PA‐BSA solution, PA was dissolved in ethanol and mixed with fatty acid‐free BSA (2% w/v in water; Bovogen Biologicals) at 37°C for 2 h with shaking. In all experiments, the dissolution solvent was used as the vehicle control, and all chemicals were purchased from Sigma‐Aldrich.

### 2.5 Animal model experiment

To evaluate protein levels in a high‐fat diet, we used liver tissue from a previously published animal model experiment.^27^ In brief, 8‐week‐old C57BL/6N male mice were obtained from Orient Bio (Sungnam, Korea) and acclimatized for at least 1 week before the experiment (24°C ± 0.5°C and 60% humidity under a 12 h light/dark cycle). The mice were fed ad libitum with either a 60 kcal% fat diet (*n* = 8) or a 10 kcal% chow diet (*n* = 8) for 8 weeks. The animals were cared for in accordance with Korea National Institute of Health guidelines, and the experiment was approved by the Institutional Animal Care and Use Committee of the Korea Institute of Science and Technology (Approval NO.: KIST‐2019‐011).

### 2.6 RNA extraction and real‐time polymerase chain reaction

Total RNA was extracted using the RNeasy Mini Kit (Qiagen) according to the manufacturer's instructions. cDNA was prepared using SuperScript III reverse transcriptase (Thermo Fisher Scientific), and quantitative real‐time polymerase chain reaction (PCR) was performed in 96‐well plates (20 μL, in triplicate) using the QuantStudio 6 Flex system (Thermo Fisher Scientific). GAPDH was used for the normalization of target gene expression, and relative quantification was performed using the 2^−ΔΔCt^ method.^28^

### 2.7 Protein extraction and Western blot analysis

Whole‐cell lysates were prepared using RIPA buffer (Sigma) with a protease inhibitor (Sigma) and sonicated according to the manufacturer's instructions. The samples were centrifuged at  18 390 *g* for 20 min at 4°C and the supernatants were used for Western blot analysis as described previously.^29^ Whole‐cell lysates (20 μg) were mixed with 20 μL of SDS‐PAGE sample buffer and loaded onto 4%–12% gels. The immunoblots were visualized using an enhanced chemiluminescence system (Thermo Fisher Scientific Inc.) and quantified using TINA 2.0 software (Raytest GmbH). Antibodies against GRP78 (sc‐13968), PEPCK (sc‐32879), and secondary anti‐rabbit and anti‐mouse antibodies were purchased from Santa Cruz Biotechnology. Plasminogen (ab154560) was purchased from Abcam. mGluR5 (GRM5) (#55920) and GAPDH (#2218) were purchased from Cell Signaling Technology.

### 2.8 ELISA

Human ELISA kits, including PAI‐1 (BMS2033, Thermo Fisher Scientific) and plasminogen (ab108893, Abcam), were used according to the manufacturer's instructions. Briefly, MSG‐treated cell culture media were harvested via centrifugation at 2122 *g* for 10 min at 4°C to remove debris. The resulting supernatants were collected and used in the experiments.

## 3 RESULTS

### 3.1 General characteristics of the subjects based on glutamate levels

The characteristics of the 1844 subjects without diabetes at baseline based on glutamate level tertiles are shown in Table S1. Mean baseline glutamate levels for T1, T2, and T3 were 109.6, 151.6, and 238.4 μm, respectively, and there were significant differences among groups. In the group with high glutamate levels, the proportion of men and smokers was high, whereas individuals classified into the T2 and T3 groups had significantly higher age and body mass index than those in T1. In particular, although all subjects were within the normal glucose tolerance range at baseline, the mean baseline fasting glucose levels at T2 and T3 were significantly higher than those at T1.

### 3.2 Association of baseline glutamate levels with diabetes incidence

The incidence of diabetes in individuals in the T1, T2, and T3 groups during the 8 years under consideration; that is, 2005–06 to 2013–14, was 74 (11.9%), 125 (20.5%), and 144 (23.5%), respectively (Table S1). Kaplan–Meier curves for diabetes events showed that the group with high glutamate levels had a higher probability of developing diabetes over 8 years (log‐rank test, *p* < .0001; Figure S2). The mean time to incident diabetes in the T3 group was 77.5 months, which was shorter than the time of 81.6 months in the T1 group. A significant increase in the hazard ratio (HR) for incident diabetes was observed in T2 (HR: 1.45; confidence interval [CI]: 1.08, 1.95) and T3 (HR: 1.63; CI: 1.22, 2.18) compared to the T1 group over the 8 years under study (Table 1). However, there were no significant aggressive effects of high glutamate levels on the incidence of diabetes when the baseline fasting glucose level was included as a covariate in the analysis. Thus, plasma glutamate levels are highly correlated with glucose levels and can work together in the development of diabetes.

**TABLE 1:** Association of baseline glutamate levels with the incidence of diabetes.

	T1	T2	T3	*p* for trend
Model1	Reference	1.74 (1.30–2.31)	2.05 (1.55–2.71)	<.0001
Model2	Reference	1.45 (1.08–1.95)	1.63 (1.22–2.18)	.0042
Model3	Reference	1.20 (0.90–1.61)	1.28 (0.96–1.72)	.2516

*Note*: Values are expressed as HR (CI). The HR (CI) was calculated using Cox proportional hazard analysis with adjustment for covariates. Model 1, no adjustment; Model 2, adjusted for baseline age, sex, body mass index, and smoking status; Model 3, Model 2 + baseline fasting glucose. Abbreviations: CI, confidence interval; HR, hazard ratio.

### 3.3 Screening for glutamate‐related genetic variants

To screen for glutamate‐related genetic variants, GWAS for log‐transformed glutamate levels was performed using a linear regression additive model. Examination of the top 30 single‐nucleotide polymorphisms (SNPs) that were associated with glutamate levels indicated that SNPs located mainly in *FRMD4B* (rs35609328, rs1532525, rs1532524), *PLG* (rs1084659, rs783184), and *PARD3* (rs1148243, rs1148242, rs2244099, rs920515) were significantly associated with glutamate (Table S2).

### 3.4 Alteration of PLG by insulin resistance

**FIGURE 1:**
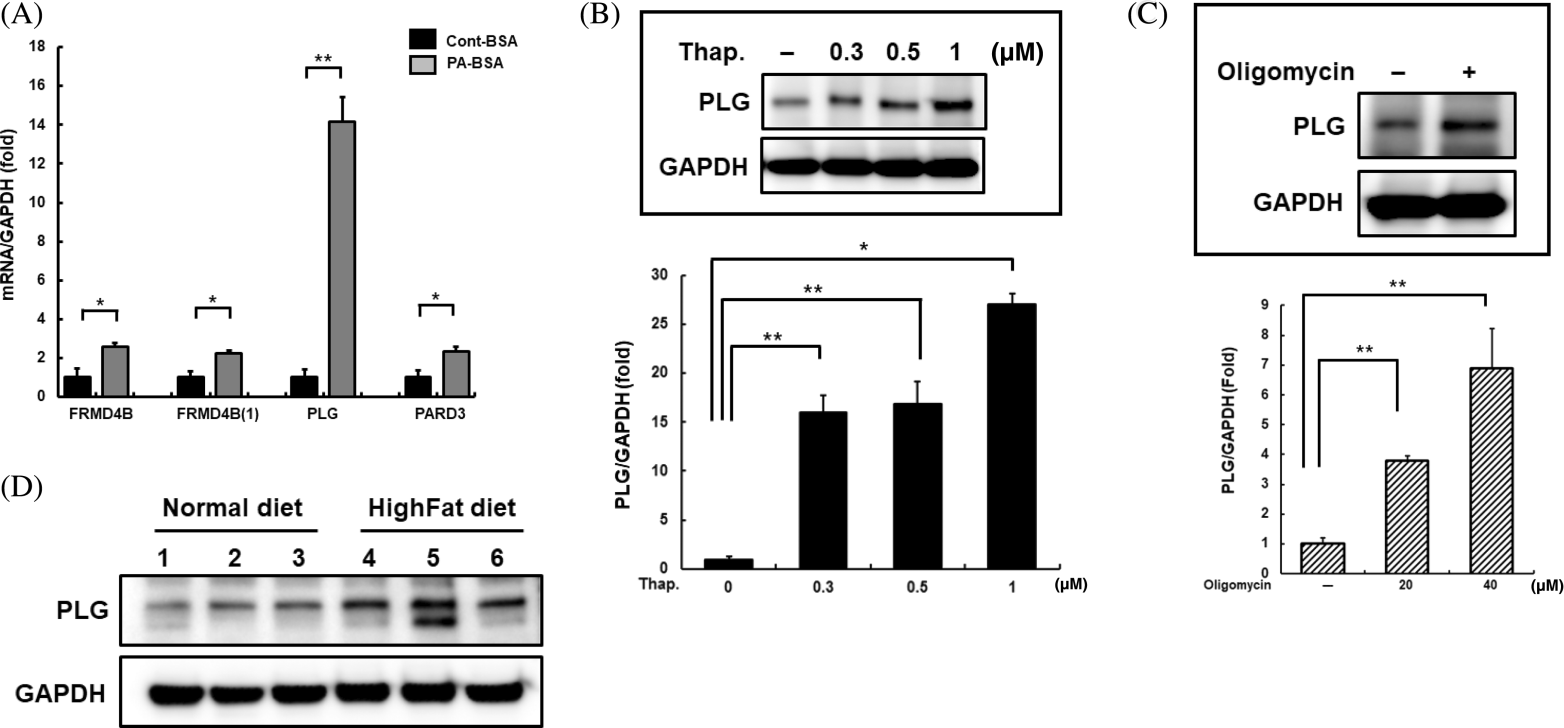
Insulin resistance elevates plasminogen levels. (A) Transcriptional levels of glutamate‐related genes (*FRMD4B*, *PLG*, *PARD3*) were analyzed in SK‐Hep‐1 cells treated with palmitate (PA; 24 h, 500 μM) via real‐time polymerase chain reaction. (B and C) The expression of plasminogen was analyzed in whole‐cell lysates of SK‐Hep‐1 cells treated with thapsigargin (24 h; 0, 0.3, 0.5, or 1 μM) or oligomycin (24 h; 20 or 40 μM) by western blotting. (D) Whole lysates were extracted from liver tissues of C57BL6 mice fed a high‐fat diet and age‐matched mice fed a standard diet, and plasminogen levels were measured by western blotting. *Significant differences between groups at *p* < .05. **Significant differences between groups at *p* < .001. PA‐BSA, palmitate‐BSA; PLG, plasminogen.

To identify the regulatory factors associated with glutamate‐related diabetes onset, the levels of *FRMD4B*, *PLG*, and *PARD3*, identified via GWAS, were evaluated for insulin resistance. We used a palmitate‐treated cell model that is typically used to study insulin resistance to verify the association with the three identified genes.^30^ Among these, the PLG mRNA level was elevated by palmitate in SK‐Hep‐1 cells (Figure 1A).

We further investigated the alteration in PLG expression after the induction of endoplasmic reticulum (ER) stress and mitochondrial dysfunction, which are closely related to insulin resistance.^31^, ^32^ Thapsigargin and oligomycin induce ER stress and mitochondrial dysfunction, respectively, resulting in insulin resistance. The protein and mRNA levels of PLG were increased by thapsigargin and oligomycin treatment (Figure 1B,C). In addition, the expression of PLG in the liver tissue of C57BL6 mice, in which insulin resistance was induced by a high‐fat diet, was significantly increased compared to that in the control group (Figure 1D). Therefore, insulin resistance‐related diabetes onset is associated with high PLG levels.

### 3.5 Association of glutamate on insulin resistance

**FIGURE 2:**
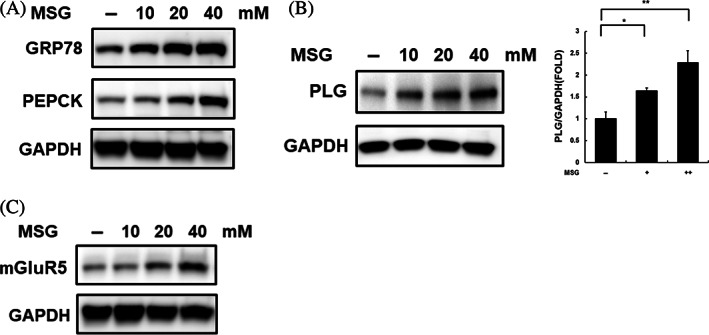
Excessive glutamate‐induced insulin resistance elevates plasminogen levels. (A) Protein expression associated with the endoplasmic reticulum stress response and gluconeogenesis was analyzed in whole‐cell lysates of SK‐Hep‐1 cells treated with monosodium glutamate (MSG; 24 h; 0, 10, 20, or 40 mM) by western blotting. (B and C) Expression of plasminogen and mGluR5 (metabotropic glutamate receptor 5) were analyzed in whole‐cell lysates of SK‐Hep‐1 cells treated with MSG (24 h; 0, 10, 20, or 40 mM) by western blotting. *Significant differences between groups at *p* < .05. **Significant differences between groups at *p* < .001. GRP78, glucose‐related protein 78; PEPCK, phosphoenolpyruvate carboxykinase; PLG, plasminogen.

Based on the clinical data analysis results, the levels of insulin resistance‐related factors were assessed in SK‐Hep‐1 cells treated with MSG to evaluate the association between glutamate and insulin. Similar to ER stress, abnormal gluconeogenesis induced insulin resistance.^33^ GRP78 (ER stress factor) and PEPCK (gluconeogenesis factor) levels increased in a dose‐dependent manner (Figure 2A). These results indicate that glutamate is closely related to insulin resistance.

Next, PLG levels were analyzed after treatment with glutamate to evaluate the effect of glutamate on PLG expression. As shown in Figure 2B, the protein and mRNA levels of PLG gradually increased in an MSG dose‐dependent manner in SK‐Hep‐1 cells for 24 h. mGluR5, a group I metabotropic glutamate receptor expressed in the liver, is associated with β‐cell dysfunction, and an increase in mGluR5 levels induces ER stress and DNA damage.^34^, ^35^, ^36^ We observed an alteration in mGluR5 expression by glutamate, and treatment with MSG resulted in a gradual increase in mGluR5 expression (Figure 2C). This finding suggests that glutamate is related to the induction of insulin resistance and an increase in PLG and mGluR5 levels in the liver.

### 3.6 Association of mGluR5 and PLG with insulin resistance

**FIGURE 3:**
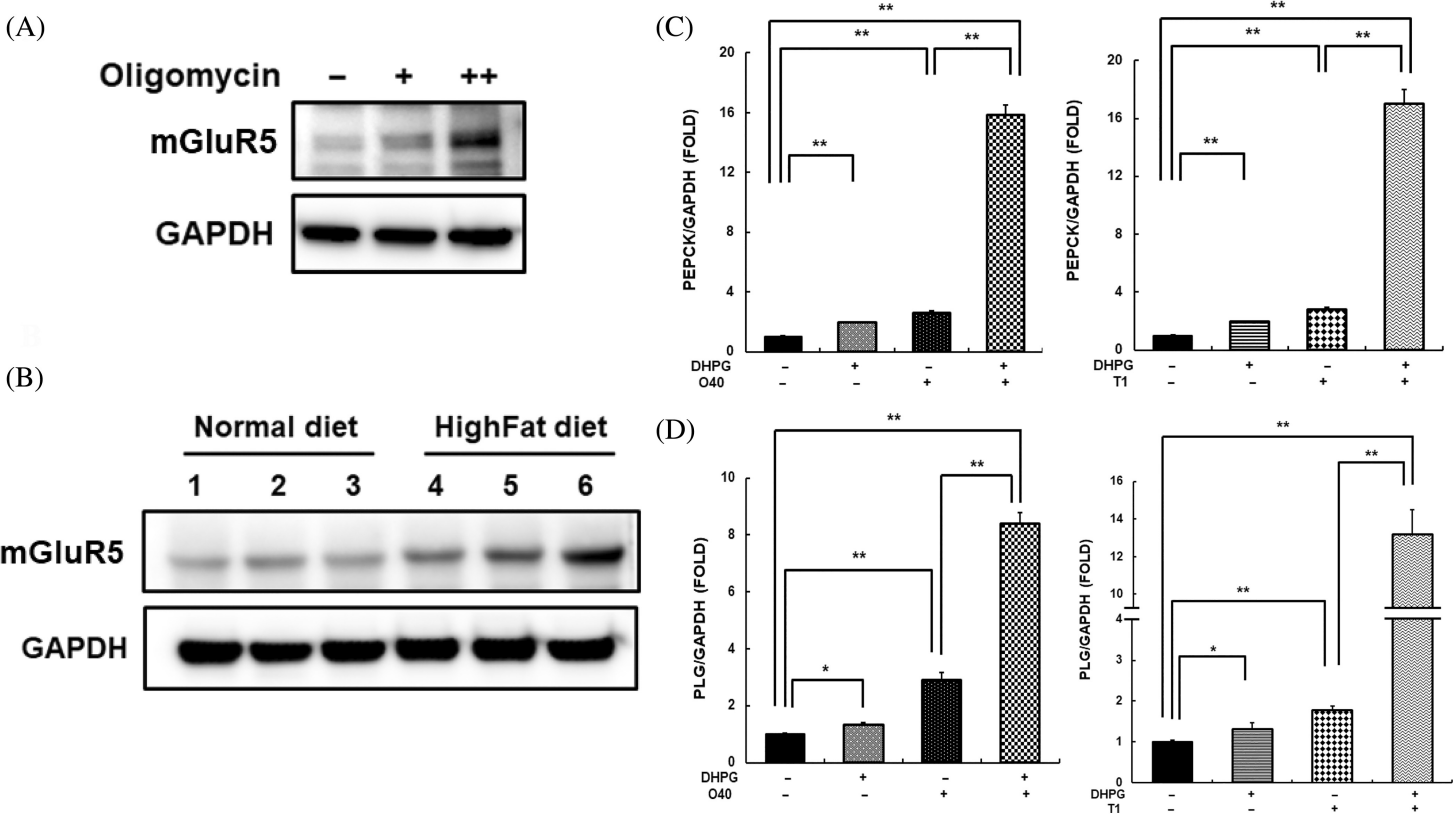
mGluR5 activation increased plasminogen levels during insulin resistance. (A) The expression of mGluR5 was analyzed in whole‐cell lysates of SK‐Hep‐1 cells treated with oligomycin (24 h; 20 or 40 μM) by western blotting. (B) Whole lysates were extracted from liver tissues of C57BL6 mice fed a high‐fat diet and age‐matched mice fed a standard diet, and mGluR5 levels were analyzed via western blotting. (C and D) The transcriptional level of PEPCK (gluconeogenesis marker) and plasminogen were assessed in SK‐Hep‐1 cells pretreated with DHPG (2 h, 20 μM), and subsequently treated with oligomycin (24 h, 40 μM) or thapsigargin (24 h, 1 μM) by real‐time polymerase chain reaction. *Significant differences between groups at *p* < .05. **Significant differences between groups at *p* < .001. DHPG, 3,5‐dihydroxyphenylglycine; mGluR5, metabotropic glutamate receptor 5; PEPCK, phosphoenolpyruvate carboxykinase.

To investigate the association between insulin resistance and mGluR5, we evaluated mGluR5 expression change in cells and animal models of induced insulin resistance. The expression of mGluR5 was increased in SK‐Hep‐1 cells treated with oligomycin (Figure 3A). The expression of mGluR5 in the liver tissue of C57BL6 mice with high‐fat diet‐induced insulin resistance was significantly increased compared to the control group (Figure 3B). These results indicated that insulin resistance is associated with high mGluR5 levels.

Additionally, mGluR5 was activated to confirm its relationship with insulin resistance. SK‐Hep‐1 cells were treated with DHPG, an mGluR5 agonist, to confirm the level of PEPCK. PEPCK mRNA levels were increased by DHPG, and combination treatment with thapsigargin or oligomycin resulted in a higher increase in PEPCK mRNA levels (Figure 3C). Furthermore, mGluR5 activation by DHPG caused increases in PLG expression, and combinatorial treatment with thapsigargin or oligomycin significantly increased PLG expression (Figure 3D). The data indicate that mGluR5 activation is associated with insulin resistance and increased PLG expression.

### 3.7 Alteration in PLG secretion and PAI‐I in glutamate‐induced insulin resistance

**FIGURE 4:**
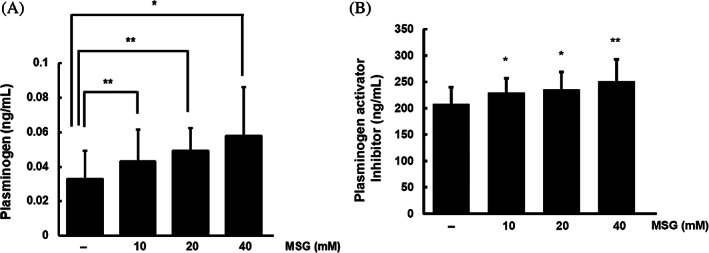
Excessive glutamate increases plasminogen levels through the regulation of plasminogen activator inhibitor levels. (A and B) The levels of secreted plasminogen and plasminogen activator inhibitor were measured in supernatants of SK‐Hep‐1 cells treated with monosodium glutamate (MSG; 24 h; 0, 10, 20, or 40 mM) via ELISA. *Significant differences between groups at *p* < .05. **Significant differences between groups at *p* < .001.

We showed that high glutamate levels induced insulin resistance, which subsequently increased the expression of PLG in the liver (Figures 1 and 2). PLG is mainly synthesized in the liver, secreted from cells, and converted by plasminogen activator (tPA) to plasmin, a fibrinolytic enzyme. Therefore, to evaluate insulin resistance induced by glutamate, we analyzed the changes in extracellular secreted PLG. Secreted PLG levels increased in a dose‐dependent manner in MSG‐treated SK‐Hep‐1 cells (Figure 4A).

PAI‐1, a tPA inhibitor, is increased in the presence of insulin resistance and diabetes, and inhibits the conversion of PLG to plasmin.^11^ We analyzed the level of secreted PAI‐1 in MSG‐induced insulin resistance and found that MSG treatment upregulated extracellular PAI‐I levels (Figure 4B). The results indicate that glutamate‐induced insulin resistance is associated with an increase in extracellular PLG and PAI‐1 levels.

## 4 DISCUSSION

As the prevalence of diabetes continues to increase worldwide, early diagnosis and treatment before onset are very important. In this study, we showed that glutamate is associated with glucose levels and diabetes development. Through GWAS analysis, *PLG* genes that were significantly associated with glutamate were identified, and the effects of glutamate and PLG on diabetes were investigated via functional studies using cell and mouse models.

Metabolic diseases, such as diabetes, show disease‐related metabolic abnormalities even before clinical symptoms appear, along with an increase or decrease in metabolic products. Metabolic and meta‐analysis studies have shown that the increase in glutamate and branched‐chain amino acids, such as leucine, isoleucine, and valine, are closely related to type 2 diabetes risk.^14^ In particular, plasma glutamate levels showed a positive correlation with glucose and insulin metabolism, and were associated with insulin resistance and increased risk of type 2 diabetes.^37^ Long et al.^38^ reported that glutamate levels were significantly different in type 2 diabetes and prediabetes stages, and that they can serve as metabolite markers that can distinguish between prediabetes and diabetes. Similar to previous studies, we observed that the group with high glutamate levels in the present study showed a significantly greater increase in diabetes incidence after 8 years than did the group with the lowest levels; moreover, the average time for diabetes onset was shorter in the former group. These results indicate that glutamate is closely related to the onset of diabetes, suggesting that glutamate levels can be considered a strong candidate biomarker for the early diagnosis of diabetes.

In previous functional studies, excessive glutamate levels were closely related to insulin resistance and diabetes, causing ER stress and β‐cell dysfunction in the pancreas.^39^, ^40^, ^41^ In addition, hyperglycemia was induced in MSG‐induced diabetic mice by encoding key gluconeogenesis enzymes.^42^ In functional studies using cell models, we observed that high concentrations of glutamate increased the ER stress and gluconeogenesis marker levels in the liver, suggesting that glutamate is associated with insulin resistance. Excessive glutamate can induce ER stress and disrupt the insulin signaling system, causing abnormal glucose production. Cohort studies have further shown that glutamate levels are significantly correlated with hepatic insulin resistance and fibrosis.^40^ These results suggest that excessive glutamate may induce insulin resistance and impact the risk of developing diabetes.

Insulin resistance and diabetes are closely related to hypofibrinolysis.^8^ Reportedly, the limitation of fibrinolysis in diabetes occurs through various mechanisms. Some of these may be caused by the reduced binding of plasminogen and tPAs to fibrin, reduced generation of plasmin, and increased PAI‐1, a potent inhibitor of fibrinolysis.^6^, ^8^ PLG is a protease that initiates fibrinolysis and is activated during inflammatory reactions, contributing to the resolution of inflammatory reactions.^43^ In diabetic patients, PLG is important because structural changes, deficiencies, and restrictions on PLG activation can lead to blood clots.^6^, ^8^

We identified glutamate‐associated genes using GWAS analysis, and PLG was identified as a regulator of glutamate‐related diabetes onset. PLG levels were increased in liver cells and mouse liver tissues induced by insulin resistance, and also in those treated with glutamate. This result is closely related to PLG and glutamate‐induced insulin resistance. Further, insulin resistance in patients with diabetes increases fibrinogen concentrations, and an increase in fibrinogen can partly activate PLG.^8^, ^44^ Therefore, our results indicate that insulin resistance induced by excessive glutamate can contribute to PLG activation, a major factor in fibrinolysis, and PLG is involved in the onset of glutamate‐related diabetes.

Glutamate works in various ways through specific glutamate receptors (iGluRs and mGluRs). Huang et al. showed that glutamate level increase can contribute to diabetes development through excessive activation of N‐methyl‐D‐aspartate receptors in β‐cells, and can accelerate apoptosis induced by β‐cell dysfunction and hyperglycemia.^34^ Further, increased glutamate levels in diabetes excessively activate mGluR5, leading to the loss of retinal function and cell death.^21^ We observed that the glutamate‐induced increase in PLG in liver cells was caused by the activation of mGluR5. Thus, excessive glutamate induces insulin resistance through the activation of mGluR5 in liver cells and contributes to an increase in PLG levels.

An increase in PLG levels was positively associated with the activation of fibrinolysis. However, insoluble fibrin cannot be broken down after increased PLG is released from the cell if it is not converted to plasmin by PAI‐1, an inhibitor of tPA. Therefore, the increase in PAI‐1 levels attenuates fibrinolysis activity in the blood.^10^ There is a close association between insulin resistance and increased PAI‐1 levels. Increased levels of insulin and its precursors (proinsulin and its split products) during insulin resistance improved the expression of PAI‐1, and this change was also observed in patients with type 2 diabetes.^41^, ^45^ Moreover, glutamate induces the synthesis and release of PAI‐1 in platelets.^46^ Similarly, in our study, an increase in extracellular secreted PLG and PAI‐1 levels was observed in glutamate‐induced insulin resistance. We are yet to analyze additional hypofibrinolytic mechanisms; however, PLG levels in cells are increased by excessive glutamate, and its transition to plasmin by PAI‐1 after being released from the cell is inhibited. Presumably, extracellular PLG continues to increase. Our data indicate that glutamate‐induced insulin resistance is involved in the hypofibrinolysis observed in type 2 diabetes by increasing the expression of PAI‐1.

In summary, our findings show that glutamate can serve as a biomarker for insulin resistance‐related diabetes onset and contributes to diabetes by inhibiting fibrinolysis. In addition, the identification of a novel mechanism for glutamate‐induced metabolic abnormality may contribute to the reduction in cardiovascular risk by normalizing fibrinolysis through glutamate signaling‐dependent target regulation.

## AUTHOR CONTRIBUTIONS

All authors had full access to the data. Hye‐Ja Lee was responsible for the integrity of the data and the accuracy of the analyses. Hyo Jung Lee, Jeong Won Yeom, Ji Ho Yun, and Hye‐Ja Lee conceived and designed the study. Han Byul Jang, Min‐Gyu Yoo, and Hyo‐Jin Kim performed the statistical analyses, and Hyo Jung Lee and Ji Ho Yun acquired and analyzed the data. All authors interpreted the data. Jeong Won Yeom wrote the initial draft of the manuscript, which was reviewed and edited by Hyo Jung Lee, Ji Ho Yun, Han Byul Jang, Soo Kyung Koo, and Hye‐Ja Lee. All authors have read and approved the final manuscript.

## CONFLICT OF INTEREST STATEMENT

The authors declare the existence of no competing interests.

## Supporting information


Appendix S1.
