# Supplementary material for: Increased glutamate in type 2 diabetes in the Korean population is associated with increased plasminogen levels
Source: J Diabetes. 2023 Jun 14;15(9):777–86. doi: 10.1111/1753-0407.13429 (PMC10509517; doi:10.1111/1753-0407.13429)
Supplement: Supplementary file 1 — Appendix S1. [file JDB-15-777-s001.docx]

**Supplementary Tables**

**Table S1. General characteristic of the subjects according to glutamate levels.**

|  | T1 | T2 | T3 | P-value |
| --- | --- | --- | --- | --- |
|  | (n=620) | (n=610) | (n=614) |  |
| Glutamate (㎛) | 109.6 ± 16.3 ^c^ | 151.6 ± 11.6 ^b^ | 238.4 ± 86.0 ^a^ | <0.0001 |
| Age | 55.0 ± 8.9 ^b^ | 58.0 ± 8.6 ^a^ | 57.1 ± 9.0 ^a^ | <0.0001 |
| Sex | 36.0/64.0 | 46.2/53.8 | 55.4/44.6 | <0.0001 |
| BMI (kg/m^2^) | 23.7 ± 3.0 ^b^ | 24.5 ± 3.2 ^a^ | 24.8 ± 3.4 ^a^ | <0.0001 |
| Glucose (mg/dL) | 87.7 ± 9.7 ^b^ | 91.5 ± 10.1 ^a^ | 92.5 ± 10.9 ^a^ | <0.0001 |
| SMOKE |  |  |  |  |
| Non-smoker | 455 (73.4) | 391 (64.1) | 329 (53.7) | <0.0001 |
| Current smoker | 98 (15.8) | 99 (16.2) | 121 (19.7) |  |
| Ex-smoker | 67 (10.8) | 120 (19.7) | 163 (26.6) |  |
| Incident DM | 74 (11.9) | 125 (20.5) | 144 (23.5) | <0.0001 |
| Follow-up months | 81.6 ± 23.0 ^a^ | 79.9 ± 23.9 ^ab^ | 77.5 ± 25.2 ^b^ | 0.0125 |

BMI, body mass index

Values are expressed as the mean ± SD or n (%); Differences among groups were assessed by general linear models. Duncan post-hoc test was used to identify group differences (a: highest mean; c: lowest mean: a>b>c)

**Table S2. Top 30 SNPs associated with glutamate levels.**

| CHR | SNP | BP | A1 | BETA | STAT | GENE | Region | P |
| --- | --- | --- | --- | --- | --- | --- | --- | --- |
| 1 | rs10799702 | 21821880 | G | 0.06 | 4.98 |  |  | 6.65E-07 |
| 3 | rs35609328 | 69517351 | C | 0.08 | 4.97 | FRMD4B | intron | 7.09E-07 |
| 2 | rs188946930 | 30702103 | T | 0.16 | 4.95 | LCLAT1 | intron | 8.04E-07 |
| 9 | 9:9520298:T_TA | 9520298 | I | 0.21 | 4.81 |  |  | 1.62E-06 |
| 3 | rs1532525 | 69516524 | G | 0.07 | 4.78 | FRMD4B | intron | 1.83E-06 |
| 3 | rs1532524 | 69516857 | G | 0.07 | 4.76 | FRMD4B | intron | 2.05E-06 |
| 11 | rs334240 | 98632634 | A | -0.06 | -4.65 |  |  | 3.42E-06 |
| 12 | rs10845389 | 11767146 | A | 0.11 | 4.60 |  |  | 4.37E-06 |
| 18 | rs4798703 | 8881276 | C | -0.05 | -4.60 |  |  | 4.44E-06 |
| 5 | rs374098 | 24459843 | T | 0.07 | 4.59 |  |  | 4.60E-06 |
| 5 | rs433145 | 24459850 | A | 0.07 | 4.59 |  |  | 4.60E-06 |
| 1 | rs10799699 | 21820961 | C | 0.05 | 4.59 |  |  | 4.74E-06 |
| 1 | rs10799700 | 21820968 | G | 0.05 | 4.59 |  |  | 4.74E-06 |
| 1 | rs10799701 | 21820990 | A | 0.05 | 4.59 |  |  | 4.74E-06 |
| 4 | rs10007944 | 181293818 | T | 0.06 | 4.57 | LOC105377567 | intron | 5.00E-06 |
| 6 | rs1084659 | 161163777 | A | 0.07 | 4.57 | PLG | intron | 5.09E-06 |
| 6 | rs783184 | 161163074 | A | 0.07 | 4.57 | PLG | intron | 5.11E-06 |
| 1 | rs1780324 | 21821757 | A | 0.05 | 4.56 |  |  | 5.26E-06 |
| 1 | rs1697422 | 21821897 | T | 0.05 | 4.56 |  |  | 5.26E-06 |
| 7 | rs78611673 | 49567364 | A | 0.19 | 4.56 |  |  | 5.36E-06 |
| 10 | rs1148243 | 34441297 | T | -0.06 | -4.56 | PARD3 | intron | 5.42E-06 |
| 8 | rs10087730 | 102139995 | T | -0.07 | -4.54 | LOC105375674 | upstream | 5.77E-06 |
| 7 | rs79955954 | 11919963 | T | -0.09 | -4.54 |  |  | 5.86E-06 |
| 10 | rs1148242 | 34441217 | T | -0.06 | -4.54 | PARD3 | intron | 5.94E-06 |
| 10 | rs2244099 | 34437623 | T | -0.06 | -4.54 | PARD3 | intron | 6.01E-06 |
| 10 | rs1148241 | 34440780 | C | -0.06 | -4.53 | PARD3 | intron | 6.07E-06 |
| 11 | rs1727149 | 95497171 | T | 0.04 | 4.53 |  |  | 6.11E-06 |
| 11 | 11:95499768:TCAAT | 95499768 | D | 0.04 | 4.53 |  |  | 6.18E-06 |
| 10 | rs920515 | 34439852 | A | -0.06 | -4.53 | PARD3 | intron | 6.26E-06 |
| 15 | 15:95436822:TA_T | 95436822 | D | 0.06 | 4.52 |  |  | 6.35E-06 |

**Supplementary Figures**


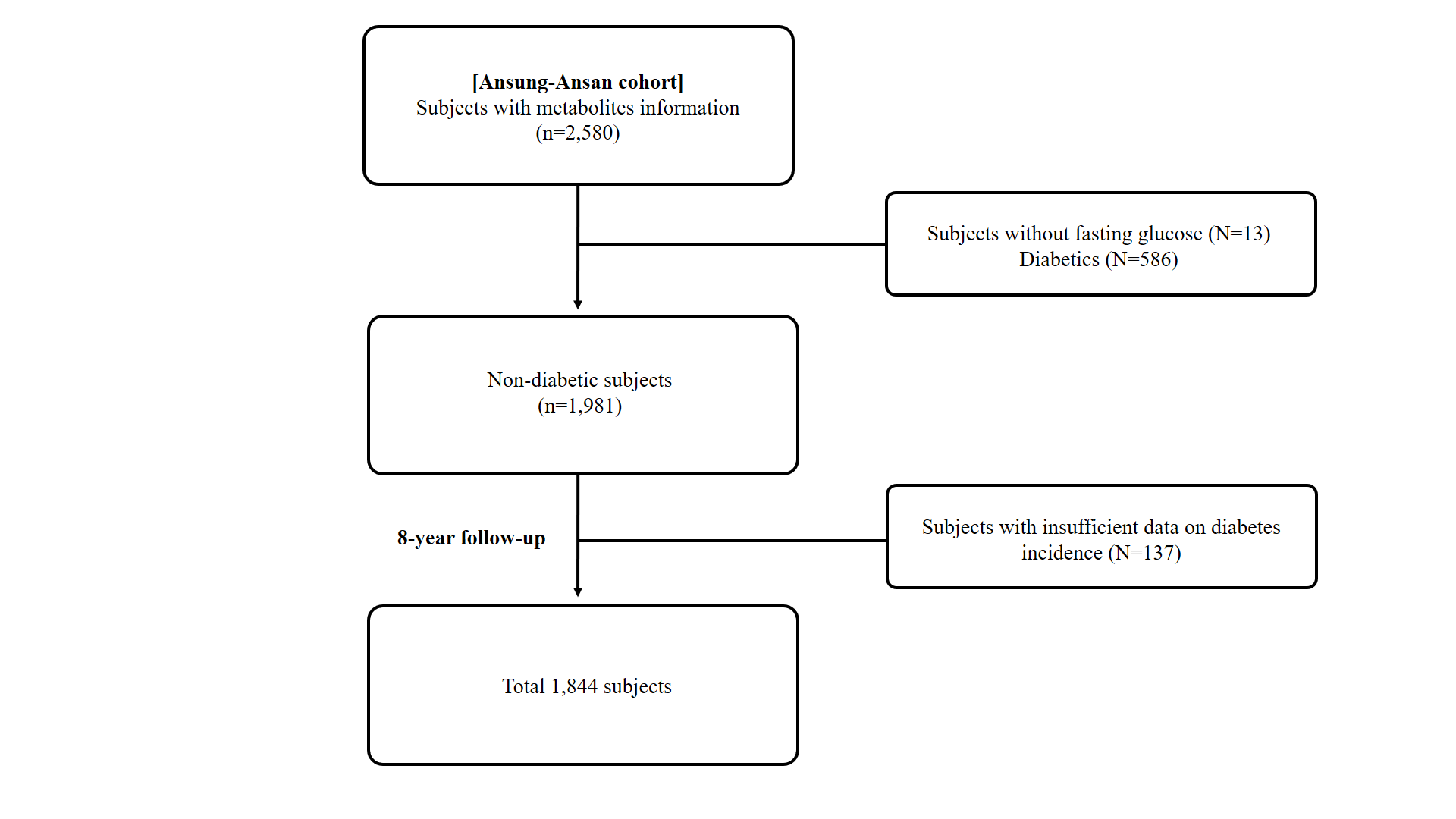


**Figure S1.** Flow chart

**
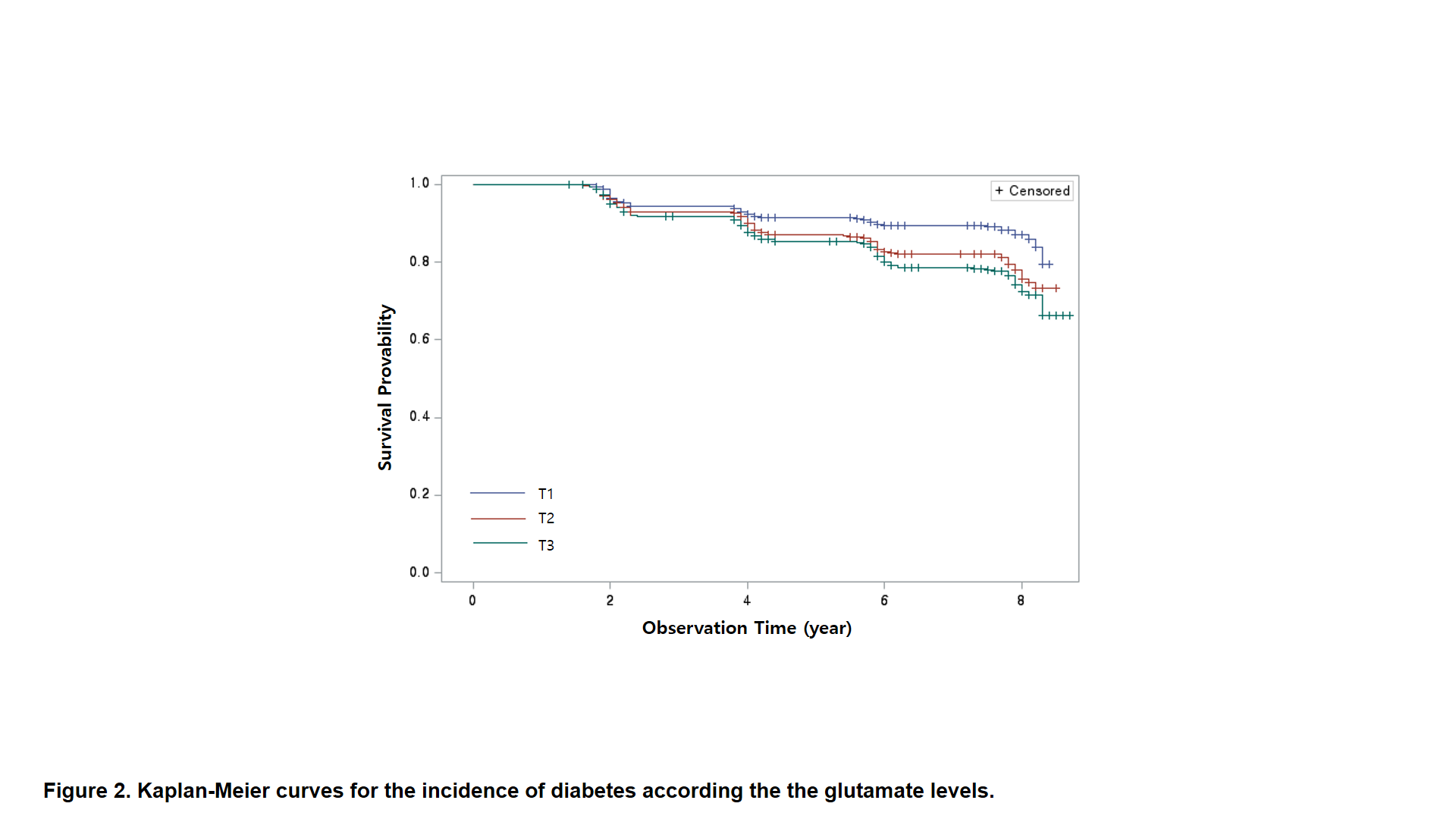
**

**Figure S2.** Kaplan-Meier curves for the incidence of diabetes based on glutamate levels.
